# Evaluation of Cytotoxic and Genotoxic Effects in Buccal Mucosal Cells in Non-Smokers and Users of Traditional Combustible Tobacco Products and Non-Combustible Alternatives

**DOI:** 10.3390/jox14010009

**Published:** 2024-01-12

**Authors:** Antonija Tadin, Vinka Stazic, Nada Galic, Davor Zeljezic

**Affiliations:** 1 Department of Restorative Dental Medicine and Endodontics, Study of Dental Medicine, School of Medicine, University of Split, 21000 Split, Croatia; 2 Department of Maxillofacial Surgery, Clinical Hospital Centre Split, 21000 Split, Croatia; 3 Health Center of Split-Dalmatia County, 21000 Split, Croatia; vinka.stazic@dz-sdz.hr; 4 Department of Endodontics and Restorative Dental Medicine, School of Dental Medicine, University of Zagreb, 10000 Zagreb, Croatia; ngalic@sfzg.hr; 5 Division of Toxicology, Institute for Medical Research and Occupational Health, 10000 Zagreb, Croatia; dzeljezic@imi.hr

**Keywords:** buccal mucosa, cytotoxicity, cytogenetic, e-cigarette, genotoxicity, heated tobacco products, micronucleus assay, smoking

## Abstract

Aims/Objectives: The aim of this cross-sectional observational study was to investigate cytogenetic damage to the buccal mucosa in non-smokers and consumers of traditional combustible tobacco products and non-combustible alternatives. Methods: A total of 160 participants were divided into four groups according to the type of product used, including non-smokers, users of conventional combustible tobacco (cigarettes), heated tobacco, and electronic, tobacco-free vapor products (e-cigarettes). Buccal mucosa samples were analyzed using the micronucleus cytome assay to assess cytotoxic and genotoxic damage. Results: E-cigarette users showed significantly higher values for all tested parameters in the micronucleus test compared to non-smokers (*p* < 0.05). Similarly, users of tobacco heating products showed an increase in all parameters (*p* < 0.05), with the exception of the number of cells with micronuclei. Conventional cigarette smokers showed a notable increase in the number of binucleated cells and cells with karyorrhexis and karyolysis (*p* ≤ 0.05). When assessing the differences between users of traditional combustible tobacco products and non-combustible alternatives, these did not appear to be significant, except for e-cigarette users, who had significantly more cells with condensed chromatin (*p* ≤ 0.001), while users of tobacco heating products had more pyknotic cells (*p* ≤ 0.001). Conclusion: The results of this study underscore the heightened occurrence of cytotoxic and genotoxic damage in users of both conventional combustible tobacco products and non-combustible alternatives compared to non-smokers, emphasizing the detrimental impact of these products on the oral mucosa.

## 1. Introduction

The global consumption of tobacco and nicotine products, both combustible and non-combustible varieties, remains a major public health challenge and continues to have complex effects on human well-being [1,2]. The combustion of tobacco products, including cigarettes, cigars, and pipes, produces smoke, exposing the tobacco to temperatures of up to 900 °C. Tobacco smoke contains a variety of chemicals, many of which are harmful and carcinogenic, posing a significant health risk with over 7000 chemical substances, including 70 known carcinogens. The chemical composition of various cigarette constituents is influenced by factors such as the type of tobacco, smoking behavior, and the characteristics of the combustion product, underscoring the importance of understanding these variables in assessing health outcomes [3].

In the area of tobacco consumption, more and more innovative tobacco and nicotine products are being advertised, as they are supposedly less harmful to health. Electronic cigarettes (e-cigarettes) change the smoking experience by using batteries to heat nicotine-enriched e-liquids and create an inhalable aerosol. In this process, the e-liquid comes into contact with a heating coil in a chamber and reaches temperatures of 100 to 250 °C. E-cigarettes were developed to replicate the nicotine experience of conventional cigarettes while minimizing the health risks. They are attractive for various reasons, such as the different flavors, the absence of smoke and odor, the elegant design, and the high nicotine concentration. Despite the intended harm reduction, the toxicity of e-cigarette aerosols depends on the intrinsic toxicity of the e-liquid and the chemicals produced during vaporization. Assessments show similarities with conventional cigarettes, which contain nicotine, nitrosamines, carbonyl compounds, heavy metals, and particulate matter, albeit in smaller quantities [4,5]. Tobacco heating products (THPs) are electronic devices that heat processed tobacco without combustion, producing an aerosol that contains fewer pollutants than cigarette smoke. THPs consist of a charger, a holder, and tobacco sticks or capsules and heat tobacco sticks at 200 to 400 °C, creating an inhalable aerosol without combustion. These “safer” heated tobacco products deliver nicotine while minimizing tar and carbon monoxide emissions [6,7], preserving the nicotine and eliminating many hazardous ingredients found in conventional cigarette smoke.

In view of the information presented, more and more research results indicate that non-combustible alternative products show a remarkable reduction in harmful substances in the smoke produced compared to conventional cigarettes. Numerous studies show a significant decrease in toxic chemicals and toxins emitted by tobacco heating products and e-cigarettes compared to conventional cigarettes [2,8,9]. Furthermore, empirical evidence from in vitro and in vivo clinical studies suggests that the biological activity of these innovative tobacco products is significantly reduced compared to the profound effects of cigarette smoke. These results make an important contribution to the ongoing discourse on harm reduction strategies for the health effects of tobacco use [8,9,10].

Despite this promise, the oral mucosa and gingival epithelium, as the human tissues in first contact with cigarette smoke and directly exposed to it, face direct consequences, including an increased risk of cancers of the oral cavity and pharynx and deleterious effects on gum tissue, contributing to an increased prevalence of periodontal disease. This causal relationship between tobacco or nicotine use and oral malignancies is supported by a comprehensive range of clinical and epidemiologic studies [11,12]. Although industry data suggest a possible attenuation of the respiratory damage associated with e-cigarettes and THPs, it is important to recognize that the majority of these studies relate primarily to short-term effects. The overarching and lasting effects of e-cigarette and heated tobacco product use on human health, particularly in the long term, remain uncertain. This prevailing ambiguity stresses the need for further comprehensive surveys and studies based on long-term public health consequences [13,14,15,16].

When evaluating the potential harm reduction of innovative tobacco products, it is essential to consider the effects on oral mucosal cells, which represent the first site of contact between the smoke or aerosol and the organism, being a sort of primary barrier against substances introduced by swallowing or inhalation. The micronucleus test, which is known for its simplicity and informative value, has proven to be a suitable instrument for such assessments, especially for tobacco products. In vitro studies have shown marked differences in cytogenetic damage to oral mucosal cells in both non-smokers and smokers, with correlations found between this damage, the duration of smoking, and the type of cigarettes used [17,18,19]. However, the limited direct comparisons between different types of tobacco products emphasize the need for further scientific investigation [20,21,22]. Given the remarkable heterogeneity of results from extensive research, further investigation is essential to promote a nuanced and comprehensive understanding of this complex topic. While the cytogenetic potential of changes in conventional cigarettes is well known, studies on the genotoxicity of e-cigarettes and THPs provide contradictory results. Contradictory results in studies may be due to different study designs, different product formulations, limited long-term data, population differences, evolving product characteristics, the interaction of different variables, and possible publication bias. This emphasizes the urgency of further research to unravel the intricate interplay between innovative tobacco products and oral mucosal health [9,10,22,23,24,25].

The aim of this in vivo study was to investigate the effects of conventional combustible tobacco products (cigarettes) and non-combustible alternatives, including e-cigarettes and heated tobacco products, on buccal mucosal epithelial cells. The primary objectives were as follows: (a) to compare the incidence of micronuclei and other nuclear abnormalities between non-smokers and users of conventional combustible tobacco products and non-combustible alternatives; and (b) to assess differences in the incidence of these nuclear abnormalities between individuals using conventional combustible cigarettes and e-cigarettes and heated tobacco products. In addition, the study investigated the influence of lifestyle habits on the occurrence of cytogenetic damage in the buccal mucosa. Hence, it is known that genomic damage can originate from various sources, including environmental exposures to genotoxins, medical procedures, micronutrient deficiencies, and lifestyle [26].

## 2. Materials and Methods

The aim of this cross-sectional observational study was to evaluate cytotoxic and genotoxic damage in exfoliated buccal mucosal cells from users of conventional combustible tobacco products and non-combustible alternatives as well as from non-smokers using the micronucleus cytome assay. The study was conducted in July and August 2021 at the Department of Restorative Dentistry and Endodontics, Faculty of Medicine, University of Split, Croatia, in compliance with all ethical standards and the Declaration of Helsinki. Ethical approval was obtained from the Ethics Committee of the Faculty of Medicine, University of Split (Approval No. 003-08/21-03/0003, Registration No. 2181-198-03-04-21-0074).

### 2.1. Participants

Each participant voluntarily agreed to take part in the study and was informed in detail about the purpose of the study before giving written consent. The study included 160 participants, consisting of 61 men (38.2%) and 99 women (61.8%) aged between 20 and 71 years (mean age 41.6 ± 14.6).

A comprehensive medical and dental history was taken from all participants. Respondents completed a questionnaire designed for this study, which recorded demographic factors (age and gender) and lifestyle habits (diet, including frequency of meat, vegetable, fruit, and alcohol consumption).

The study comprised four different groups of participants, which differed according to their self-reported smoking status: non-smokers, users of traditional combustible cigarettes, e-cigarettes, and heated tobacco products. Crucially, when categorizing conventional combustible tobacco products—cigarettes—the study did not consider the manufacturer or the nicotine content of the products used by the participants. Furthermore, even in users of non-combustible alternatives, details such as the manufacturer, nicotine content, type of flavor, and other additives were not taken into account. Moreover, participants were not queried about prior usage of other products currently under research, and whether they were dual users of the examined products. The selection of participants was based on predefined inclusion and exclusion criteria. Inclusion criteria necessitated a minimum of one year of using the researched products, voluntary consent, adulthood, classification under ASA I according to the American Society of Anesthesiology, the absence of prosthetic and orthodontic replacements in the oral cavity, the absence of precancerous lesions, no periodontal diseases, no radiation exposure in the head and neck area, and no use of antibiotics, corticosteroids, or bisphosphonates in the past six months. Conversely, exclusion criteria encompassed systemic diseases, and pregnancy or lactation in women. Respondents whose samples could not be processed or who did not provide complete information in the survey were also excluded.

To determine the required minimum sample size, we utilized the sample size effect (Cohen’s d), derived from Upadhyay et al. [27]. Based on the difference in micronuclei occurrence in buccal mucosa cells between non-smokers (1.03 ± 1.27) and smokers (7.58 ± 5.67), the sample size effect (Cohen’s d) was calculated as 1.594. Using a significance level of α = 0.05 and 80% power for the test, we determined that a minimum of 16 subjects per group should be included in the study. To address the potential loss of participants during the study, the sample size was expanded to include 40 respondents.

### 2.2. Cell Sampling

Epithelial cell samples from the buccal mucosa were obtained from each subject using the brushing technique. An hour prior to the sampling the participants withhold from consuming any food or drinks besides water. Before sampling, participants were instructed to rinse their mouths thoroughly with water. After rinsing with tap water, cytological samples were collected using a brush (Cytobrush Plus, GmbH, Dietramszell-Linden, Germany). The brush was gently applied to the buccal cheek mucosa and a swab was taken. The collected cells were evenly spread on labeled slides, air-dried, and then fixed in methanol and glacial acetic acid solution (in a 3:1 ratio) at 4 °C for at least 20 min. Staining of the slides followed the procedure described by Thomas et al. [28].

The analysis was carried out using an Olympus CX 40 light microscope (Olympus, Tokyo, Japan) at 400× magnification, with some observed anomalies further scrutinized at 1000× magnification. Each sample was prepared in duplicate and placed on separate slides, and 2000 epithelial cells were examined per person (1000 cells per slide). The occurrence of nuclear abnormalities (micronucleus, binucleated cell, karyorrhexis, karyolysis, pyknosis, nuclear bud, and ‘broken egg’) was assessed and categorized according to the method described by Tolbert et al. [29].

### 2.3. Statistical Analysis

The collected data were entered into an existing table using Microsoft Excel 2007 (Microsoft Corporation, Redmond, Washington, DC, USA) and then coded for further analysis in the Statistical Package for the Social Sciences (SPSS, version 26, IBM Corp, Armonk, NY, USA). An initial analysis of the results was conducted using descriptive statistics. Categorical data were shown as absolute numbers (n) and percentages (%), whereas continuous data were shown as mean ± standard deviation (SD) or median (interquartile range). The normality of data distribution was determined with the Shapiro–Wilk test. Group differences were compared using Kruskal–Wallis’s analysis of variance. When the post hoc test was required, the Bonferroni correction was performed. Multiple regression analysis was used to evaluate the influence of predictor variables (such as age, gender, and dietary habits) on dependent variables (including micronucleus count, binucleated cells, nuclear bud cells, pyknotic cells, cells with condensed chromatin, karyolytic cells, and cells with condensed chromatin). All statistical analyses were conducted with a predetermined significance level of *p* < 0.05.

## 3. Results

A total of 160 subjects participated in the research, 40 in each group. Among the respondents, those who consumed heated tobacco products had the shortest consumption experience, while those who smoked classic combustible cigarettes had the longest consumption experience, as summarized in Table 1.

The results of the micronucleus test are presented in Table 2. The Kruskal–Wallis test indicated statistically significant variations among the groups using tobacco products in different forms concerning all cytogenetic parameters examined. When compared to non-smokers, individuals who used traditional combustible tobacco products (cigarettes) exhibited a noteworthy increase in the number of binucleated cells (*p* ≤ 0.001) and the number of cells with karyorrhexis (*p* ≤ 0.001) and karyolysis (*p* ≤ 0.001). Contrary to non-smokers, e-cigarette users showed significantly higher values for all tested parameters in the micronucleus cytome assay compared to non-smokers (*p* < 0.05). Similarly, users of tobacco heating products showed an increase in all parameters (*p* < 0.05), with the exception of the number of cells with micronuclei.

While assessing disparities among users of various tobacco products, the majority of these distinctions seem to lack significance, except for a few noteworthy exceptions. Between users of different tobacco products, no statistically significant differences were observed in biomarkers of DNA damage, including the number of cells with micronuclei (*p* = 0.099) and the number of cells with nuclear buds (*p* = 0.937). Likewise, no significant differences were found in the cytokinesis effects (binucleated cells, *p* = 0.449). However, statistically significant differences were detected in two types of cell death lesions, the number of cells with condensed chromatin (*p* < 0.001) and pyknosis (*p* < 0.001), whereas the number of cells with karyolysis (*p* = 0.970) and karyorrhexis (*p* = 0.106) showed no significant difference. E-cigarette users exhibited a higher number of cells with condensed chromatin (*p* ≤ 0.001) compared to traditional cigarette smokers. Users of heated tobacco products displayed a significantly increased number of cells with pyknosis (*p* < 0.001) when compared to traditional smokers.

**Figure 1:**
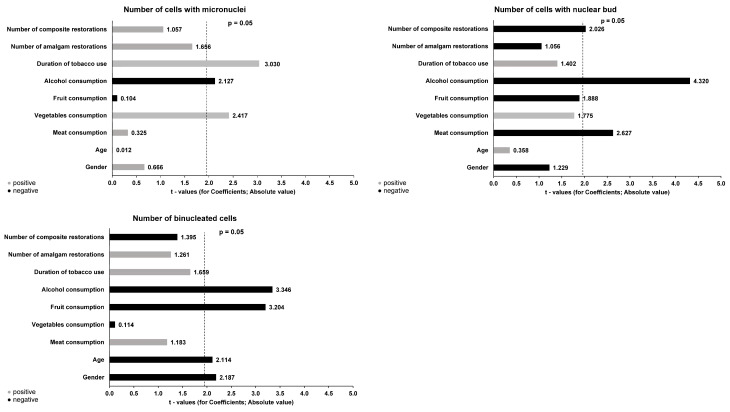
Multiple regression analysis of predictive factors for cytogenetic parameters (number of cells with micronuclei, nuclear buds, and binucleated cells) in buccal mucosa.

**Figure 2:**
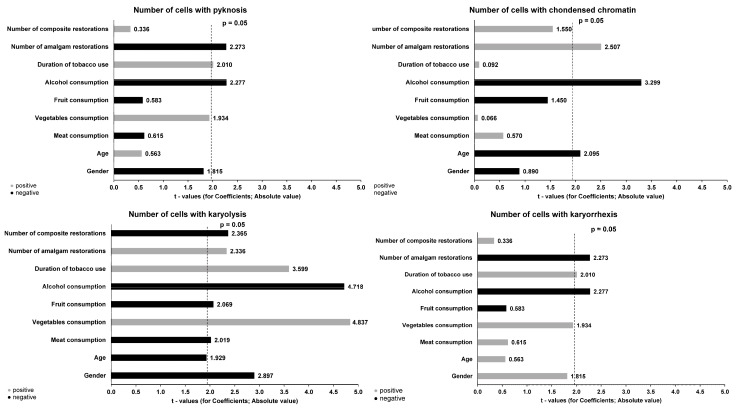
Multiple regression analysis of predictive factors for cytogenetic parameters (number of cells with karyolysis, karyorrhexis, pyknosis, and condensed chromatin) in buccal mucosa.

Figure 1 and Figure 2 present the results of the multiple regression analysis, which explores the association between the number of cytogenetic effects and demographic and lifestyle factors as potential predictive variables. Regarding the number of cells with micronuclei, a statistically significant positive correlation was observed with vegetable consumption (β = 0.244, *p* = 0.017) and smoking duration (β = 0.045, *p* = 0.003), and a negative correlation with alcohol consumption (β = −0.147, *p* = 0.035).

## 4. Discussion

The main objective of this cross-sectional study was to investigate the potential DNA damage in buccal mucosa cells in non-smokers and users of traditional combustible tobacco products and non-combustible alternatives. To assess genotoxicity and cytotoxicity, the buccal micronucleus cytome assay was used, a reliable method capable of accurately detecting nuclear damage under both in vivo and in vitro conditions [30,31]. The results of the study showed a statistically significant decrease in the occurrence of all tested cytogenetic parameters analyzed in non-smokers compared to the groups of traditional combustible tobacco products and non-combustible alternative product users. It is noteworthy that, in general, no statistically significant differences in biomarkers for DNA damage and cytokinesis effects were found between the different groups of different tobacco product users. However, statistically significant differences were detected in two types of cell death lesions. Users of conventional cigarettes exhibited a lower occurrence of cells with condensed chromatin and pyknosis compared to users of alternative products.

Tobacco is an extreme example of a systemic mutagen in humans, with 4-aminobenzene being the most potent [31,32]. Therefore, many studies have indicated the deleterious effects of the consumption of various tobacco products on the cells of the oral cavity [12,33,34]. The buccal mucosa, which is easily accessible for sampling, may contain genetic damage in the form of micronuclei or other nuclear abnormalities that are induced in the basal layer stem cells under tobacco exposure and later manifest as nuclear abnormalities in the cells scraped [35]. Users of traditional combustible tobacco products (cigarettes) and non-combustible alternatives exhibited an increase in the number of cells with a micronucleus compared to non-smokers, with only e-cigarette users reaching statistical significance. Numerous studies have consistently confirmed the increased prevalence of micronuclei in the buccal epithelial cells of smokers compared to non-smokers [18,19,36,37,38,39,40]. In addition, several studies have shown that the total number of micronuclei increases significantly with the duration of smoking, the frequency of smoking, and the type of cigarettes used [18,19,39,40]. The longer and more frequently individuals indulge in tobacco use, the more pronounced the cumulative effect of carcinogens becomes, leading to increased genotoxicity, as shown by the increased number of micronuclei [39]. In addition, the study by Naderi et al. provides empirical evidence that cigarettes can cause chromosomal damage even in the early stages of consumption [18]. In contrast, Nersesyan et al. considered not only the duration and frequency of smoking but also the nicotine and tar content of cigarettes [17]. Their results showed a significant increase in the number of cells with micronuclei, especially in the subgroup that consumed the strongest type of cigarettes, which had a nicotine content of 1.6 to 1.7 mg and a tar content of 23 to 26 mg. These disparities in findings may be attributed to variations in the levels of tar and nicotine in the cigarettes consumed by the study participants.

Micronuclei may not only be observed in buccal cells but may also indicate a broader systemic genotoxicity associated with exposure to tobacco products and affecting different cell types [30]. This was illustrated in a study conducted by Burgaz et al. in 1995, which documented a significantly increased incidence of cells with micronuclei in exfoliated urothelial cells in individuals who smoked conventional cigarettes [41]. In addition to a statistically significant difference between the control group of non-smokers and smokers overall (*p* < 0.001), a distinction was noted among subjects who smoked more than 20 cigarettes per day compared to those who smoked half as many, though this effect did not reach statistical significance. A similar study focusing on cervical cells in vivo was conducted by Nersesyan et al. [42], who found an increased number of micronuclei in smokers, but this correlated with the age of the women studied. In particular, a statistically significant increase in the prevalence of cells with micronuclei was observed in postmenopausal women, a phenomenon that can be attributed to the cumulative accumulation of mutagens from tobacco over time.

This study showed a significant increase in the prevalence of binucleated cells and cells with karyorrhexis and karyolysis in conventional cigarette smokers compared to non-smokers. The descriptors observed in the buccal micronucleus test indicate a significant effect of tobacco on the proliferation potential of the mucosal cells of the oral cavity, which mainly affects apoptosis [29]. Indicators of cellular demise and cytokinesis impairment were similarly substantiated in the buccal cells of tobacco users by Nersesyan [17] and Najeeb [43] and their co-researchers. In contrast to the results of this study, the same changes in female smokers were not identified as statistically significant in the study conducted in Iran, and no differences were found depending on the duration of smoking [39]. However, the authors used Papanicolaou staining, which is less sensitive in detecting chromatin aberrations as reported by Thomas et al. [28] in addition to additional nuclear aberrations.

In comparison to non-smokers, individuals using e-cigarettes displayed a statistically significant increase in all examined cytotoxic/genetic parameters. These increased cytotoxicity and genotoxicity parameters suggest that electronic cigarettes do not come without risk to the buccal cells, though being unjustifiably promoted as less harmful. Moreover, in contrast to conventional cigarette smokers, e-cigarette users exhibited higher counts of cells with condensed chromatin, indicating a potentially more significant cytotoxic effect on the buccal mucosa. Yu et al. [44] assessed the cytotoxic and genotoxic effects of e-cigarettes on the oropharynx. Employing in vitro testing, they encountered the effect in either healthy epithelial cells alongside head or neck squamous cell carcinoma cell lines. The study findings indicated a noteworthy reduction in cell viability and clonogenic survival in both tested cell groups exposed to e-cigarettes, regardless of nicotine content. A potential limitation of this investigation is the fact that the experiment has been performed in vitro, and that carcinoma cells already exhibiting genotypic impairments are considered. However, by having them evaluated, significant information on the effect of smoking on cells already being immortalized and transformed has been obtained. In a divergence from the aforementioned in vitro investigation, Franco et al. [45] undertook an in vivo study focusing on cells of the oral mucosa. Their findings led to the conclusion that, in comparison to traditional cigarettes, e-cigarettes represent a comparatively safer option. In that study, it was confirmed that the prevalence of micronuclei was significantly decreased in the e-cigarette smoker group in comparison to smokers. It is crucial to underscore that, despite the exclusive use of e-cigarettes by our study participants for a minimum of one year, the potential influence of their previous consumption of traditional cigarettes on the obtained results cannot be definitively ruled out. Similar to our study, a study from Brazil found that e-cigarette users had a higher number of cells with a nuclear bud, karyolysis, and binucleation compared to non-smokers. In comparison to the smokers of traditional cigarettes, only an increase in binucleated cells was observed [22].

Recent systematic reviews have indicated that heated tobacco presents a comparatively lower public health concern and a reduced risk of chronic diseases in contrast to traditional cigarettes [15,46]. While in vitro studies support the notion that heated tobacco is potentially less harmful than conventional cigarettes [9,10], this study, specifically comparing heated tobacco product (HTP) with traditional cigarette users, revealed a heightened incidence of cells with pyknosis in the heated tobacco product group. This finding suggests a potentially more pronounced cytotoxic effect compared to conventional cigarettes. It is essential to acknowledge, however, that heated tobacco devices had the shortest duration of use among the tested groups, and these observed changes might be influenced by participants’ previous habits of smoking traditional cigarettes. Upon comparing users of heated tobacco products and e-cigarettes, no statistically significant difference in other nuclear anomalies between these two groups was observed. Given that heated tobacco is a relatively new product compared to traditional e-cigarettes, there may not be enough existing studies to provide a comprehensive basis for comparing the obtained results.

The reasons for the increased parameter of cytogenetic damage, despite evidence from numerous studies demonstrating lower concentrations of hazardous compounds in the mainstream smoke of e-cigarettes and heated tobacco products compared to combusted traditional cigarettes, can be attributed to the heating temperatures and the temperature of the inhaled and exhaled air [2,16,47,48,49,50].

This study observed the impact of sociodemographic and dietary habits on the incidence of cytogenetic damage across all subjects. Notably, female gender exhibited a negative correlation with an increased occurrence of cells with karyolysis (β = −0.199, SE = 0.499, *p* = 0.004). As anticipated, the duration of tested product usage had a positive correlation with the number of cells with micronucleus (β = 0.342, SE = 0.015, *p* = 0.003), karyolysis (β = 0.292, SE = 0.054, *p* = 0.003), karyorrhexis (β = 0.352, SE = 0.032, *p* = 0.002), and pyknosis (β = 0.235, SE = 0.031, *p* = 0.046). Numerous studies have consistently confirmed an elevated incidence of DNA damage associated with the duration of smoking [9,18].

This study is subject to several limitations that may impact the interpretability of the results. Firstly, its cross-sectional design and the utilization of a convenience sampling method for respondents introduce inherent limitations. An additional constraint arises from the variability in the types of cigarettes used by participants across different groups, potentially originating from different manufacturers, leading to variations in the chemical composition of the products. Moreover, the duration of exposure to tobacco smoke represents another limiting factor, suggesting the need for future research to consider standardization of exposure times. Furthermore, the challenge of identifying individuals who exclusively use e-cigarettes and heated tobacco products without a history of traditional cigarette smoking poses a significant limitation, potentially influencing the obtained results. Additionally, it is crucial to note that our study did not delve into the specific types of heated tobacco products and tobacco-free electronic vaping products consumed by respondents. This omission could potentially impact the outcomes and highlight the necessity of examining differences between these products in future research. Understanding the nuances associated with various product types will contribute to a more comprehensive assessment of their potential effects. As a recommendation for future investigations, standardization of applied techniques is essential to enhance the reliability and comparability of findings.

## 5. Conclusions

Taking into account the aforementioned limitations of the study and in the context of its conditions, the results mean that the observations of the study confirm the presence of cytotoxic and genotoxic effects resulting from the use of both traditional combustible tobacco products and non-combustible alternatives on exfoliated oral mucosal cells. Non-smokers demonstrated significantly lower levels of damage compared to individuals using traditional combustible tobacco products—cigarettes—and non-combustible alternatives—heated tobacco products and e-cigarettes. No difference was found between the various products for most of the parameters tested. Notable findings include an increased occurrence of cells with pyknosis among users of heated tobacco products, while users of e-cigarettes exhibited a heightened frequency of cells with condensed chromatin compared to traditional cigarette smokers. Addressing this intricate public health issue necessitates thorough control tests and further research. Additionally, the implications of promoting alternative tobacco use methods should be carefully evaluated, particularly considering the ongoing prevalence of smoking in the population.

## Figures and Tables

**Table 1:** Sociodemographic data of respondents.

Characteristics		Non-Smokers (n = 40)	Cigarette (n = 40)	E-Cigarette (n = 40)	Tobacco Heating Product (n = 40)	Total (n = 160)
Age		47.8 ± 12.8	43.1 ± 16.3	40.1 ± 14.5	35.4 ± 12.2	41.6 ± 14.6
Gender	Male	20 (50.0)	12 (30.0)	13 (32.5)	16 (40.0)	61 (38.2)
	Female	20 (50.0)	28 (70.0)	27 (67.5)	24 (60.0)	99 (61.8)
Duration of product use (years)		0	13.3 ± 13.9	5.1 ± 3.4	2.3 ± 0.7	6.9 ± 9.4

**Table 2:** The occurrence of nuclear abnormalities in buccal mucosal cells among tested groups.

Cytogenetic Parameters		Non-Smokers	Cigarette	E-Cigarette	Tobacco Heating Product	*p* Values
Number of cells with micronuclei	Mdn (IQR)	1.00 (0.00−2.00) ^A^	2.00 (1.00−2.50)	2.00 (1.25−2.00) ^A^	1.50 (0.00−2.00)	0.011 *
	M ± SD	1.22 ± 1.00	1.95 ± 1.46	1.85 ± 0.58	1.40 ± 1.17	
Number of cells with nuclear bud	Mdn (IQR)	0.00 (0.00−1.00) ^B,C^	1.00 (0.00−1.00)	1.00 (0.00−1.00) ^B^	1.00 (0.00−1.50) ^C^	0.012 *
	M ± SD	0.43 ± 0.63	0.85 ± 0.80	0.90 ± 0.74	0.90 ± 0.81	
Number of binucleated cells	Mdn (IQR)	5.00 (3.00−5.75) ^D,E,F^	7.00 (2.25−9.00) ^D^	7.00 (5.00−9.00) ^E^	7.00 (5.00−5.00) ^F^	≤0.001 *
	M ± SD	4.50 ± 2.44	6.37 ± 3.64	7.35 ± 2.45	7.30 ± 2.90	
Number of cells with karyorrhexis	Mdn (IQR)	6.00 (4.25−7.00) ^G,H,I^	8.50 (6.00−11.00) ^G^	10.00 (8.00−12.00) ^H^	8.00 (7.00−11.00) ^I^	≤0.001 *
	M ± SD	6.25 ± 2.47	8.58 ± 3.17	9.98 ± 2.13	8.87 ± 3.24	
Number of cells with karyolysis	Mdn (IQR)	9.50 (4.25−9.50) ^J,K,L^	16.00 (10.00−18.00) ^J^	15.00 (11.00−18.00) ^K^	15.00 (13.00− 17.00) ^L^	≤0.001 *
	M ± SD	8.73 ± 3.14	14.75 ± 5.56	14.85 ± 3.54	14.88 ± 3.86	
Number of cells with condensed chromatin	Mdn (IQR)	4.00 (2.00−8.75) ^M,N^	8.00 (4.25−9.00) ^O^	10.00 (8.00−11.00) ^M,O^	8.50 (6.25−10.75) ^N^	≤0.001 *
	M ± SD	4.88 ± 3.73	7.12 ± 3.01	9.70 ± 1.63	8.80 ± 2.80	
Number of cells with pyknosis	Mdn (IQR)	4.00 (4.00−6.00) ^P,Q^	5.00 (4.25−9.00) ^R^	6.00 (4.25−8.00) ^P^	7.50 (4.50−9.00) ^Q,R^	≤0.001 *
	M ± SD	4.35 ± 1.98	5.12 ± 1.80	6.24 ± 1.99	6.90 ± 2.47	

* Within the same row, the same upper letter indicates a statistically significant difference between the examined groups, *p* ˂ 0.05 (^A^ *p* = 0.028, ^B^ *p* = 0.026, ^C^ *p* = 0.036, ^M^ *p* = 0.044, ^D,E,F,G,H,I,J,K,L,N,O,P,Q,R^ *p* ≤ 0.001). Abbreviation: M: mean; Mdn: median; IQR: interquartile range; SD: standard deviation.

## Data Availability

The data that support the findings of this study are available upon request from the authors.
